# A deep learning-based model for screening and staging pneumoconiosis

**DOI:** 10.1038/s41598-020-77924-z

**Published:** 2021-01-26

**Authors:** Liuzhuo Zhang, Ruichen Rong, Qiwei Li, Donghan M. Yang, Bo Yao, Danni Luo, Xiong Zhang, Xianfeng Zhu, Jun Luo, Yongquan Liu, Xinyue Yang, Xiang Ji, Zhidong Liu, Yang Xie, Yan Sha, Zhimin Li, Guanghua Xiao

**Affiliations:** 1 Shenzhen Prevention and Treatment Center for Occupational Diseases, Shenzhen, Guangdong China; 2 Quantitative Biomedical Research Center, Department of Population and Data Sciences, University of Texas Southwestern Medical Center, Dallas, TX 75390 USA; 3 Department of Mathematical Sciences, The University of Texas at Dallas, Richardson, TX 75080 USA; 4 Institute of Occupational Medicine of Jiangxi, Nanchang, Jiangxi China; 5 Shenzhen Association of Occupational Health, Shenzhen, Guangdong China; 6 Huizhou Prevention and Treatment Center for Occupational Diseases, Huizhou, Guangdong China

**Keywords:** Occupational health, Statistics

## Abstract

This study aims to develop an artificial intelligence (AI)-based model to assist radiologists in pneumoconiosis screening and staging using chest radiographs. The model, based on chest radiographs, was developed using a training cohort and validated using an independent test cohort. Every image in the training and test datasets were labeled by experienced radiologists in a double-blinded fashion. The computational model started by segmenting the lung field into six subregions. Then, convolutional neural network classification model was used to predict the opacity level for each subregion respectively. Finally, the diagnosis for each subject (normal, stage I, II, or III pneumoconiosis) was determined by summarizing the subregion-based prediction results. For the independent test cohort, pneumoconiosis screening accuracy was 0.973, with both sensitivity and specificity greater than 0.97. The accuracy for pneumoconiosis staging was 0.927, better than that achieved by two groups of radiologists (0.87 and 0.84, respectively). This study develops a deep learning-based model for screening and staging of pneumoconiosis using man-annotated chest radiographs. The model outperformed two groups of radiologists in the accuracy of pneumoconiosis staging. This pioneer work demonstrates the feasibility and efficiency of AI-assisted radiography screening and diagnosis in occupational lung diseases.

## Introduction

Pneumoconiosis is a major occupational lung disease with increasing prevalence and severity worldwide. The term “pneumoconiosis” summarizes all lung diseases caused by excessive exposure to dust (e.g., silica, asbestos, coal, and mixed dust), which often occurs in the workplace. It is an irreversible disease with no cure^1^. In 2013 alone, it caused approximately 260,000 deaths globally^2^. In the United States, the prevalence of coal worker’s pneumoconiosis (CWP; a major type of pneumoconiosis) has been increasing over the last decade and now exceeds 10% among long-tenured miners nationwide and 20% in central Appalachia^1^. Recently, there has been a surprising resurgence of progressive massive fibrosis, a disabling and fatal form of CWP, in central Appalachia^3,4^. Lung transplantation is also increasingly common among CWP patients^5^. According to the Centers for Disease Control and Prevention (CDC), during 1999–2016, the mean years of potential life lost attributed to CWP increased from 8.1 to 12.6 years^6^. In addition, more than one million workers in the United States are exposed to crystalline silica, which potentially leads to silicosis^7^, an irreversible, often disabling type of pneumoconiosis^8^. Furthermore, in recent years, more reports have indicated that dental technicians also suffer from the disease caused by inhalation of various airborne particles^9^. Because of the lengthy and unnoticeable progression of pneumoconiosis, and the seriousness of its outcomes, regular screening of the population at potential risk is the key to the early intervention and prevention of pneumoconiosis.

Current clinical diagnosis of pneumoconiosis is mainly based on the examination of chest radiographs (i.e. X-ray images). In 1980, the International Labor Organization (ILO) established a standardized system to classify radiographic abnormalities of pneumoconiosis according to the profusion level of small opacities observed in the lung^10^. This system has greatly facilitated pneumoconiosis screening and staging by providing a commonly accepted standard and guidelines. However, radiograph-based diagnosis of pneumoconiosis still requires a well-trained and experienced radiologist to visually identify subtle graphic patterns and features described in the ILO guidelines. This process is laborious and subject to considerable inter- and intra-observer variations. For example, the concordance in pneumoconiosis diagnosis is between 85 and 90% among expert radiologists^11^, and around 80% in general medical staff^12^ in the United States. The consistency is likely to be even worse for the screening programs conducted at community sites, especially in developing countries. Therefore, early detection and intervention of pneumoconiosis still demand a solution to timely, accurate, and efficient screening at a relatively low economic cost.

In order to improve the diagnostic efficiency and accuracy among radiologists, a variety of methods have been developed to analyze the chest radiographs. Since 1975, researchers have been studying the feasibility of computer-based classification of profusion in chest radiographs. For example, Hall et al*.*^13^ used pattern recognition techniques with spatial moments for the analysis of radiology images. Ledley et al*.*^14^ developed a texture analysis method to classify chest chest radiographs. Savol et al*.*^15^ investigated an adaptive object-growing algorithm based on image intensity in order to recognize small rounded opacities. Since 2010, with the rise of computer-aided detection (CAD), several image analysis methods for pneumoconiosis diagnosis based on texture features in chest radiographs have been proposed^16–19^. Several image analysis methods have been developed to assist radiologists by reducing their workload or improving the performance in various tasks in disease diagnosis^20–22^. However, all of these methods rely on certain level of “handcrafted” feature definition, which is technically challenging and time-consuming, especially for complex tasks^23^ like pneumoconiosis diagnosis and staging.

Recently, artificial intelligence (AI) has been making remarkable success in medical image analysis owing to the rapid progress of “deep learning” ^24–27^. For example, studies have demonstrated the feasibility of using AI for diagnosis of lung abnormalities such as lung nodules, pulmonary tuberculosis, cystic fibrosis, and pneumoconiosis^28^. AI algorithms have achieved performance comparable to radiology experts in interpreting radiographs^29^. However, there is a lack of AI-based diagnostic tools for pneumoconiosis because of the complexity of the disease and the limited availability of well-annotated pneumoconiosis chest radiographs for training AI models.

## Results

### Staging consistency and accuracy for experienced radiologists

**Figure 1:**
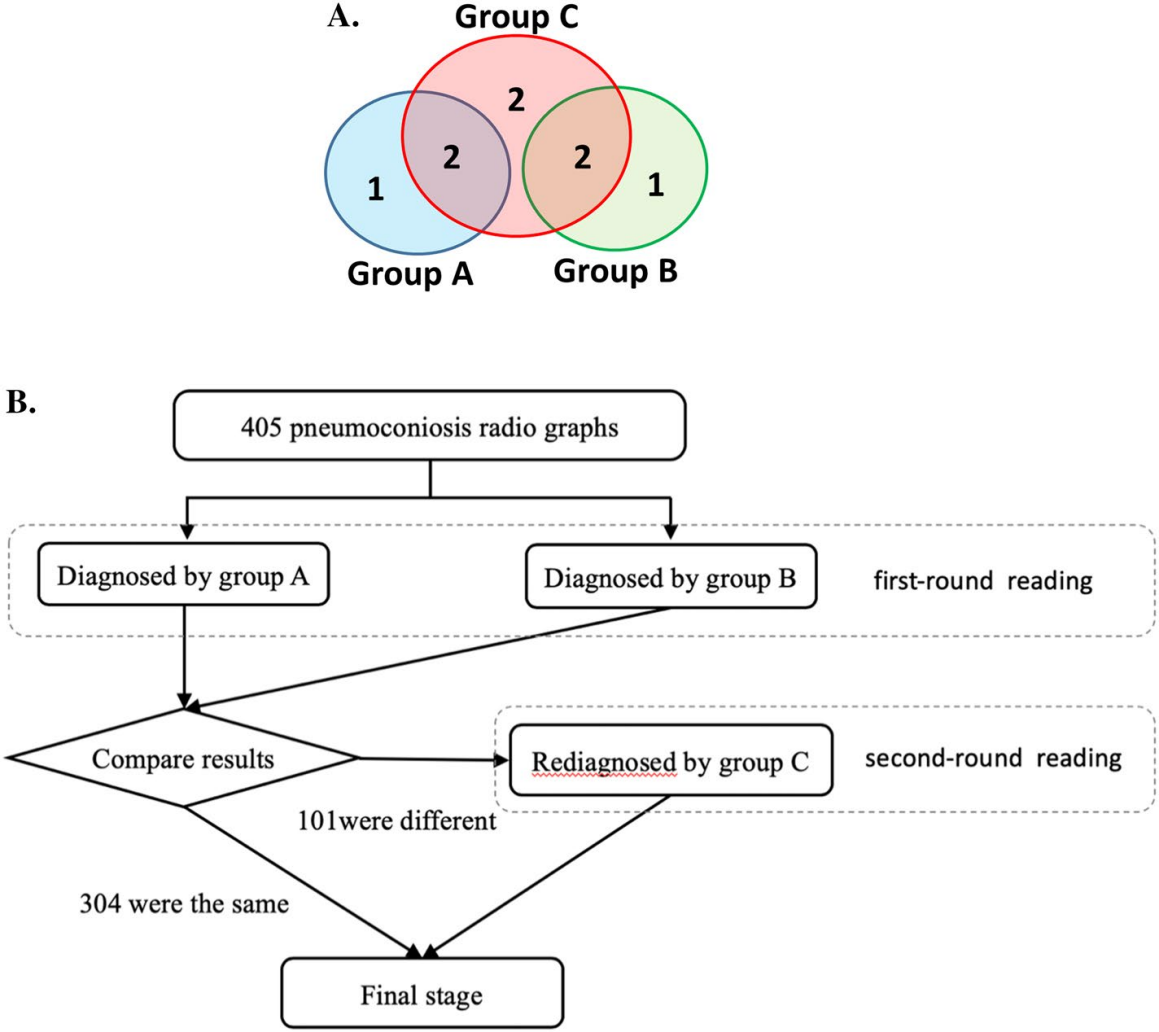
Annotation scheme. (**A**) Group assignment for radiologists. (**B**) The procedure of labelling chest radiographs in the training dataset.

For the 405 pneumoconiosis patients in the training cohort, radiologist groups A and B diagnosed the stage independently through first-round reading (with discussion only within group and not between groups). The two groups reached the same staging results for 304 patients, while differed for the remaining 101 patients. Using the final stage as the ground truth (Fig. 1B), the staging accuracy was 0.84 and 0.87 for groups A and B, respectively, which underlines the challenge of accurate diagnosis even with a group of experienced radiologists.

### Model development

The subregion-based model training process stopped at the 1200th epoch after validation accuracy failed to improve after 600 epochs. The model with the highest validation accuracy was selected as the final model.

### Model evaluation and validation

Model evaluation results using the independent test dataset are summarized in Table 1. At subregion level, the top-right, top-left, and middle-right subregions had outstanding performance in screening accuracy (> 0.9), while the other three subregions also had acceptable performance (> 0.8). The screening sensitivity of the two middle subregions were over 0.9, while that of the two bottom subregions were slightly over 0.5. For the screening specificity, all subregions had good performance (0.88–0.94).

**Table 1:** Screening and staging performance on subregion level and subject level, respectively, in the test dataset.

Subregion	Screening			Staging accuracy
	Accuracy	Sensitivity	Specificity	
Top-right	0.929	0.882	0.942	0.866
Top-left	0.912	0.829	0.930	0.864
Middle-right	0.934	0.908	0.941	0.895
Middle-left	0.881	0.918	0.883	0.842
Bottom-right	0.856	0.604	0.907	0.849
Bottom-left	0.818	0.500	0.876	0.805
Subject	0.973	0.981	0.970	0.927

At subject level, the screening accuracy was 0.973 for the independent test cohort (n = 411), with both sensitivity and specificity above 0.97 (Table 1). The staging accuracy was 0.927 (Table 1), better than that achieved by human experts (0.84 and 0.87 for radiologist group A and B, respectively). Table 2 lists the confusion matrix of the final staging results on the test dataset.

**Table 2:** Staging prediction results in the test dataset.

Predicted stage	True stage				Total
	Normal	Stage I	Stage II	Stage III	
Normal	295	2	0	0	297
Stage I	8	67	9	4	88
Stage II	0	4	12	2	18
Stage III	1	0	0	7	8
Total	304	73	21	13	411

## Discussion

According to the ILO guidelines, diagnosis and staging of pneumoconiosis are mainly based on the profusion level of small opacities and the presence of large opacities/assembly of small opacities in chest radiographs. Accurate diagnosis and staging of pneumoconiosis requires a well-trained and experienced radiologist to distinguish subtle graphic patterns in the chest radiographs. This process is time-consuming, subjective, and often results in inconsistent and unreliable outcomes, especially for pneumoconiosis screening programs implemented in underdeveloped regions. In this study, we leveraged powerful deep learning algorithms and well-annotated chest radiographs to develop and validate a deep learning-based model for screening and staging of pneumoconiosis. In order to have a set of reliable labels as the “ground truth” to train the deep learning model, we assembled a team of eight experienced radiologists and performed a two-round reading in a double-blinded fashion. Half of these radiologists have participated in developing the latest diagnosis standard of pneumoconiosis in China, which ensured the reliability of the labels.

The major differences between the proposed deep learning-based model and the other models for screening of pneumoconiosis are: (1) The deep learning model developed in our study has a higher screening accuracy, compared to the reported values in other papers. The screening accuracies in other reported studies are 0.79 (Cai et al., 2012), 0.92 (Zhu et al., 2014) and 0.94 ~ 0.95 (Yu et al., 2011), while the screening accuracy for our model is 0.97. (2) Our model not only provides a screening result (disease or not), but also the stage of the disease, which can greatly facilitate the diagnosis and treatment planning. (3) Our model for screening and staging pneumoconiosis is based on the profusion level of small opacities and the presence of large opacities in the six subregions of a lung field, which provides more enriched radiographic information for early diagnosis and intervention. (4) The data augmentation methods are widely adopted in the computer vision and deep learning field. These are the most common techniques to increase sample size and avoid overfitting in training a deep learning model^30,31^. With proper augmentation, our deep learning model gained better generalization power and achieved the same level of accuracy and stability on unseen (testing) dataset compared with the training dataset.

Due to the large size of digital chest radiographs and the often limited computing resources at the actual screening sites, it is impractical to develop and deploy a deep learning-based tool that directly works on whole chest radiographs. Existing solutions either down-sample the image (e.g., Gaussian pyramid) or reduce the model capacity (e.g., shallow learning, small CNN, etc.) to a feasible scale. However, pneumoconiosis diagnosis highly relies on identifying subtle image features, rendering not much room for downgrading the image resolution or compromising feature richness by model simplification. Alternatively, in this study, we designed a two-stage deep learning model following a divide-and-conquer strategy, which simplifies the problem into classifying the relatively small subregions in the lung instead of the whole image. This modeling strategy also mimics the clinical diagnostic procedure, which allowed us to well incorporate human knowledge (Table 3) into the AI model. It has two major advantages: (1) it largely reduces the computational load and thus the requirement of computing resources; (2) by incorporating human knowledge on pneumoconiosis staging, it reduces the sample size needed in training data.

**Table 3:** Rule-based determination of final stage according to GBZ70-2015.

	Description
Normal	No opacities discover, or Level 1 profusion of opacity presented in one subregion
Stage I	Level 1 profusion of opacities presented in more than two subregions, or Level 2 profusion of opacities presented in four subregions or less
Stage II	Level 2 profusion of opacities presented in four subregions or more, or Level 3 profusion of opacities presented
State III	Large opacities presented

In practice, pneumoconiosis screening is usually conducted at community sites, where resources, image quality and expertise level may vary. In order to improve the robustness and generalizability of the developed model with a limited amount of training data, we performed extensive image augmentation, which is one of the most effective pre-processing strategies to enrich data variety and avoid overfitting. In this study, the original images were augmented with rotating, flipping, shifting, zooming and varying the image intensity, resolution and quality. These augmentation processes largely reduced the influence of sampling condition, image contrast and lung size, and reinforced the model to focus on image features associated with pneumoconiosis.

Low accuracies were observed in the three subregions: middle-left (0.881), bottom-right (0.856), and bottom-left (0.818). This is because the middle-left region has a relatively small area due to its overlaps with the heart in a projection plane. Each of the right and left lungs has a hilum that lies roughly midway down the lung. When moving from the hilum to the periphery on the bottom, there will also be a gradual diminution of the lung markings. All these anatomical features weaken the accuracy in the two bottom subregions. In contrast, the right bronchus is relatively shorter and flatter than those in the left. As a result, inhaled dust tends to be deposited there. Thus, the top-right and middle-right subregions have more easily identifiable radiographic abnormalities in pneumoconiosis, resulting in higher accuracies.

There are several limitations in our study. Firstly, the sample size in the test dataset is relatively small. More subjects are needed, especially those with stage II and III pneumoconiosis, for a more comprehensive evaluation of the model. Secondly, the current datasets only contain chest radiographs. Although chest radiographs the standard modality for pneumoconiosis diagnosis, computed tomography (CT) typically provides more details of the opacities regions in the lung and should be tested in future studies. Lastly, the current deep learning-based model is mainly trained by those well-labeled chest radiographs from pneumoconiosis patients and healthy people. It cannot differentiate pneumoconiosis from other lung diseases that share a similar pathology. The predictive model could be further improved by (1) integrating other useful key factors to pneumoconiosis, such as working history, respiratory assessment, lungs' main function, and blood pressure, and (2) feeding more comprehensively labeled chest radiographs from patients with different lung diseases.

## Methods

### Ethics approval and consent to participate

The University of Texas Southwestern Institutional Review Board granted approval for this research. Data were collected under information consent for study participation. All methods were performed in accordance with the relevant guidelines and regulations.

### Training and test datasets

The training dataset was obtained from a cohort of 805 subjects (400 healthy controls, 163 stage I, 125 stage II, and 117 stage III pneumoconiosis patients). The test dataset was obtained from an independent cohort of 411 subjects (304 healthy controls, 73 stage I, 21 stage II and 13 stage III pneumoconiosis patients), which was used to evaluate the model developed from the training dataset. The subjects were enrolled from two different sites to ensure generalizability of the resulting model. All the enrolled chest radiographs (one image per subject) had been carefully checked for quality and graded either “Good” or “Acceptable” by radiologists, indicating no technical defects likely to impair the diagnosis and staging of pneumoconiosis.

In this study, we used the “final stage” determined by radiologists (see below) as the ground truth. To improve the diagnosis accuracy, we recruited eight experienced radiologists and divided them into three groups (Fig. 1A): three in group A and three in group B, with group C consisting of six radiologists (two from group A, two from group B, and two additional). The six radiologists in group C were selected from six different national institutions for occupational disease research in China, all of whom had more than 10 years’ experience in pneumoconiosis. Moreover, four of these radiologists participated in the development of the latest diagnostic standard of pneumoconiosis in China: “Diagnosis of occupational pneumoconiosis GBZ70-2015.”^32^.

**Figure 2:**
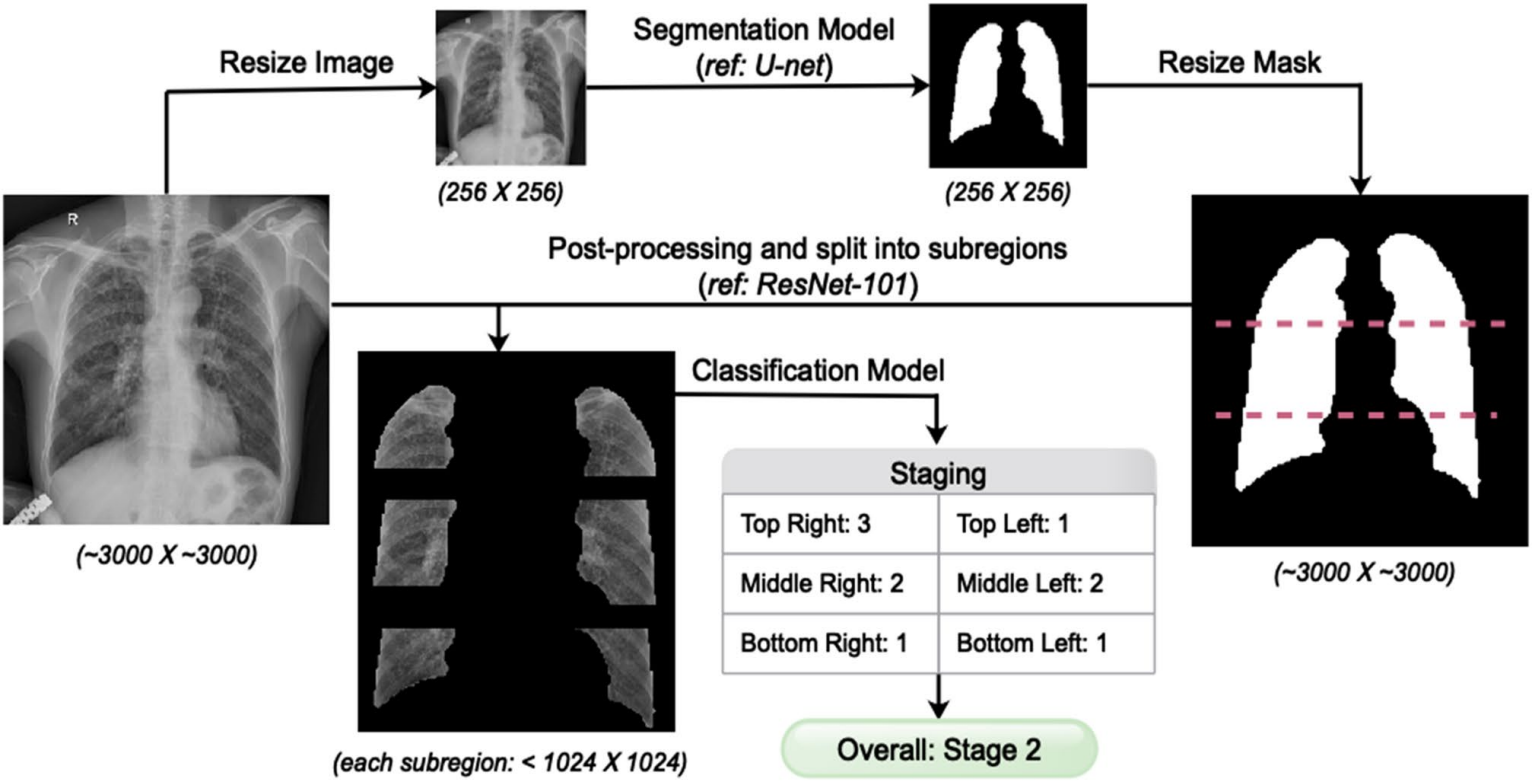
A novel two-stage deep learning model for pneumoconiosis screening and staging.

For each subject, the radiologists in group A and B independently performed the first-round reading according to the national standard, GBZ70-2015. The lung field in each chest radiographs had been divided into six mutually exclusive subregions (see “Lung field segmentation” section for details): top-right, top-left, middle-right, middle-left, bottom-right, and bottom-left (Fig. 2). Radiologist groups A and B independently annotated for each subregion the profusion level of small opacities (level 0, 1, 2, or 3) and the presence of big opacities (see Table 4 for an example record form). Then, the stage of pneumoconiosis was determined following GBZ70-2015 (Table 3). For each subject, if the staging by groups A and B agreed, it was used as the final stage. Otherwise, the radiologist group C would read the image again and determine the final stage. This procedure was applied to the entire training and test dataset (Fig. 1B).

**Table 4:** Example of record form filled by each group of radiologists for diagnosis.

Index:	001	Photograph ID:	0001	Stage:	II
Affected zones:	Right	Left		Profusion of opacity	2
Top	1	2		Assembly of small opacities or large opacities	0
Middle	2	1		Reader	A
Bottom	0	1			

### Model strategy

According to the routine diagnostic procedure for pneumoconiosis, we developed a two-stage deep learning model based on a divide-and-conquer strategy, which segmented a lung field into six subregions, trained subregion-specific models individually, and combined subregion-based predictions for staging (Fig. 2).

#### Lung field segmentation

To identify the lung field in each chest radiographs, we developed a segmentation model by adapting a U-Net semantic segmentation model together with a ResNet backbone. We first downsized the images to 256 × 256 and fed them into the model, and then resized the resulting mask image back to the original size. This resizing step allowed the model to process the images more efficiently. Then, we refined the mask with post-processing steps and extracted the lung field from the raw image (Fig. 2). To improve the accuracy and robustness of the model, we further performed extensive data augmentation, including rotating, flipping, shifting, zooming, and varying the image intensity, resolution and quality.

After segmentation, the lung field was divided into six subregions (by even distribution along the vertical direction; Fig. 2). Each subregion was only less than 1/8 of the whole image (less than 1024 × 1024), which makes the developed model feasible for the computational resources when deployed at real-world screening sites.

#### CNN for subregion classification

Next, we developed a subregion-based CNN classification model. We adapted a ResNet model to classify the profusion level of small opacities as well as the presence of large opacities for each of the six subregions in the lung field. Each subregion was classified as level 0, 1, 2, or 3 based on its opacity level. At the model development stage, the training cohort (805 subjects) was randomly separated into training (72 healthy controls, 72 stage I, 96 stage II, and 96 stage III), validation (24 healthy controls, 18 stage I, 8 stage II, and 8 stage III) and test sets (304 healthy controls, 73 stage I, 21 stage II, and 13 stage III). The developed model was finally evaluated in the independent test cohort of 411 subjects. Last, each subject was assigned into one of the four stages: normal, stage I, II, or III, by integrating the subregion-based classifications following GBZ70-2015 (Table 3).

### Statistical analyses

To evaluate the screening performance, we used three common metrics for binary classification systems: (1) accuracy (ACC_scr_, the percentage of radiographs being correctly classified as normal or disease), sensitivity (SEN, the percentage of pneumoconiosis radiographs being correctly classified as abnormal), and specificity (SPE, percentage of normal radiographs being correctly classified as normal). To evaluate the staging performance, we only used accuracy (ACC_sta_ , the percentage of radiographs being correctly classified as normal, stage I, II, or III). All statistical analyses were carried out using R version 3.6.1.

### Computing environment

In this study, computational works were conducted in a computing environment using the interface of Python 3.0 based on a TensorFlow deep learning framework, which was installed and executed on a server with Linux version 3.16.0-69-generic and Ubuntu 4.8.2-19 in 64 bits. This server also includes two Intel Xeon CPU E5-2680 v3 processors of 2.50 GHz and a 30 Mb Cache, where each processor has 12 cores and the total number of logical CPU cores is 48. The server has 132 Gb RAM and an NVIDIA Tesla K40 m GPU with 2880 stream cores, 12 Gb maximum memory, 288 Gb/s maximum memory bandwidth, and 6 GHz memory clock speed.
